# Age-dependent motor dysfunction due to neuron-specific disruption of stress-activated protein kinase MKK7

**DOI:** 10.1038/s41598-017-07845-x

**Published:** 2017-08-04

**Authors:** Tokiwa Yamasaki, Norie Deki-Arima, Asahito Kaneko, Norio Miyamura, Mamiko Iwatsuki, Masato Matsuoka, Noriko Fujimori-Tonou, Yoshimi Okamoto-Uchida, Jun Hirayama, Jamey D. Marth, Yuji Yamanashi, Hiroshi Kawasaki, Koji Yamanaka, Josef M. Penninger, Shigenobu Shibata, Hiroshi Nishina

**Affiliations:** 1 Department of Developmental and Regenerative Biology, Medical Research Institute, Tokyo Medical and Dental University (TMDU), 1-5-45 Yushima, Bunkyo-ku, Tokyo, 113-8510 Japan; 2 Laboratory of Physiology and Pharmacology, School of Advanced Science and Engineering, Waseda University, Tokyo, Japan; 3 Department of Hygiene and Public Health I, Tokyo Women’s Medical University, Tokyo, Japan; 4 Laboratory for Molecular Dynamics of Mental Disorders, RIKEN Brain Science Institute, Wako, Saitama, 3510198 Japan; 5 Center for Nanomedicine, SBP Medical Discovery Institute, Department of Molecular, Cellular, and Developmental Biology, University of California Santa Barbara, Santa Barbara, California USA; 6 Division of Genetics, Department of Cancer Biology, The Institute of Medical Science, The University of Tokyo, Shirokanedai, Minato-ku, Tokyo 108-8639 Japan; 7 Department of Medical Neuroscience, Graduate School of Medical Sciences; Brain/Liver Interface Medicine Research Center, Kanazawa University, Kanazawa, Japan; 8 Department of Neuroscience and Pathobiology, Research Institute of Environmental Medicine, Nagoya University, Nagoya, Japan; 9 IMBA, Institute of Molecular Biotechnology of the Austrian Academy of Sciences, Vienna, Austria

## Abstract

c-Jun N-terminal kinase (JNK) is a member of the mitogen-activated protein kinase family and controls various physiological processes including apoptosis. A specific upstream activator of JNKs is the mitogen-activated protein kinase kinase 7 (MKK7). It has been reported that MKK7-JNK signaling plays an important regulatory role in neural development, however, post-developmental functions in the nervous system have not been elucidated. In this study, we generated neuron-specific *Mkk7* knockout mice (MKK7 cKO), which impaired constitutive activation of JNK in the nervous system. MKK7 cKO mice displayed impaired circadian behavioral rhythms and decreased locomotor activity. MKK7 cKO mice at 8 months showed motor dysfunctions such as weakness of hind-limb and gait abnormality in an age-dependent manner. Axonal degeneration in the spinal cord and muscle atrophy were also observed, along with accumulation of the axonal transport proteins JNK-interacting protein 1 and amyloid beta precursor protein in the brains and spinal cords of MKK7 cKO mice. Thus, the MKK7-JNK signaling pathway plays important roles in regulating circadian rhythms and neuronal maintenance in the adult nervous system.

## Introduction

The c-Jun N-terminal kinases (JNKs) belong to the mitogen-activated protein kinase (MAPK) family and control diverse physiological processes including both apoptosis and cell survival^1, 2^. The JNKs consist of three related genes: *Jnk1*, *Jnk2*, and *Jnk3* ^1^. Mitogen-activated protein kinase kinase 7 (MKK7) and MKK4 are activators of JNKs in response to various stimuli such as environmental stresses, growth factors, hormones, and pro-inflammatory cytokines^1, 3, 4^. MKK7 is a specific upstream kinase of JNKs, while MKK4 activates both JNKs and another MAPK, p38^5^. The activation of JNKs leads to the phosphorylation of various substrates, including the AP-1 transcription factor c-Jun^6^.

The importance of JNK signaling in the nervous system was established by studying JNK knockout mice^7, 8^. *Jnk1* ^*−/−*^ *Jnk2* ^*−/−*^ double knockout (DKO) mice die at embryonic day 11.5 (E11.5) with failure of neural tube closure and decreased apoptosis in the hindbrain but increased apoptosis in the forebrain^9, 10^. *Jnk1* ^−/−^ single knockout mice display abnormalities in the maintenance of telencephalic commissures^11^, dendritic architecture^12^ and synaptic plasticity^13^. *Jnk2* ^*−/−*^ knockout mice display defective synaptic plasticity^14^ and resistance to neuronal cell death induced by the neurotoxin prodrug MPTP^15^ in the adult brain. *Jnk3* ^−/−^ knockout mice also show resistance to MPTP-^15^ as well as kainic acid-^16^ and ischemia-^17, 18^ induced neuronal cell death, and abnormal circadian behavioral rhythms^19^. Together, these reports indicate that JNK isoforms compliment each other but also regulate diverse roles in brain development, neuronal activity, and cell death.

To identify novel functions of JNK signaling, researchers have focused on MKK4 and MKK7 as they act as a bottleneck by regulating all three JNK isoforms. Mkk4^−/−^Mkk7^−/−^ (Mkk4/7 DKO) mice die at around E9.5 before neural tube formation^4^, and even Mkk4^−/−^ or Mkk7^−/−^ single knockout mice cause embryonic lethality at around E11.5^20–23^. *Mkk4* ^*flox/flox*^ *Nestin-Cre* mice, in which *Mkk4* was deleted specifically in neural stem cells and postmitotic neurons, died at three week-old, displaying misaligned Purkinje cells in the cerebellum and delayed radial migration and axonal degeneration^24^. *Mkk7* ^*flox/flox*^ *Nestin-Cre* mice showed severe defects in radial migration and axon elongation, and died at birth^25^. These results highlight the value of MKK4 and MKK7 for unraveling potentially novel functions of JNK signaling.

Circadian clocks are internal oscillators that drive various rhythmic activities at the cellular and organismal levels over a period of around 24 hours^26^. A self-sustained transcription/translation loop generates molecular oscillations of the core clock components, CLOCK, BMAL1, PER, and CRY^27^. The JNK inhibitor SP600125 and knockdown of *Jnk* using siRNAs lengthen the period of circadian transcription in cell culture systems^28–30^. In addition, our group revealed that *Mkk7*-deleted mouse embryonic fibroblasts showed an elongated period of circadian transcription^31^. However, the function of MKK7-JNK signaling on animal circadian behaviors has not been elucidated.

In this study, we generated neuron-specific *Mkk7* deleted mice. We found that MKK7 is essential for regulation of the circadian clock at the animal level. In addition, we discovered an unexpected role for MKK7 in motor function in adult animals.

## Materials and Methods

### Animals

Mice carrying the *Mkk7 flox* allele were described previously^32^. *Mkk7* ^*flox/flox*^ were crossed to *Synapsin-Cre* (*Syn-Cre*) transgenic mice expressing Cre recombinase under the control of the rat *synapsin* promoter^33^. We crossed male *Mkk7* ^*flox/flox*^ mice with female *Mkk7* ^*flox/+*^ *Syn-Cre* mice for generation of neuron-specific MKK7 conditional knockout mice. Because *Syn-Cre* was slightly expressed in mouse testis, resulted pups from male *Mkk7* ^*flox/+*^ *Syn-Cre* mice were haploinsufficient for MKK7 in the whole body (Fig. S1). The resulting control (*Mkk7* ^*flox/+*^, *Mkk7* ^*flox/flox*^, *Mkk7* ^*flox/+*^ *Syn-Cre*) and MKK7 cKO (*Mkk7* ^*flox/flox*^ *Syn-Cre*) mice of both sexes were used for the experiments. Mice were reared on a normal 12 h light/dark schedule. LacZ reporter mice (Gt[ROSA]26Sor^tm1Sor^, the Jackson Laboratory) were crossed with *Syn-Cre* mice and subjected to β-gal histochemical analysis. Mice genotypes were determined by PCR and Southern blotting. For all experiments, only littermate mice from the same breeding were used. All procedures were performed in accordance with a protocol approved by the Tokyo Medical and Dental University and Waseda University Animal Care Committees.

### Protein preparation and immunoblotting

Protein preparation and immunoblotting were performed as previously described with slight modifications^25^. Proteins were extracted from tissues in TNE buffer (20 mM Tris-HCl, pH 7.4, 150 mM NaCl, 1 mM EDTA, 1 mM EGTA, 0.5% Nonidet P40, 5% (w/v) glycerol, 1 mM PMSF, 200 mM NaF, 200 μM Na_3_VO_4_, 10 μg/ml aprotinin) or RIPA buffer (50 mM Tris-HCl, pH 8.0, 150 mM NaCl, 5 mM EDTA, 15 mM MgCl_2_, 1% Nonidet P40, 0.5% sodium deoxycholate, 1 mM PMSF, 50 mM NaF, 100 μM Na_3_VO_4_, 10 μg/ml aprotinin) and homogenized using a dounce homogenizer. Protein concentrations were quantified using the BCA^TM^ Protein Assay Kit (PIERCE). Protein extracts were fractionated by SDS-PAGE and transferred to a PVDF membrane, which was incubated in blocking solution (2% skim milk in TBS) for 1hr. The blocked membrane was incubated overnight in TBS containing 5% BSA plus antibodies recognizing phospho-JNK (Cell Signaling), JNK1 (Santa Cruz), MKK7 (Cell Signaling), GAPDH (Chemicon), phospho-MKK4 (Cell Signaling), MKK4 (Santa Cruz), JIP1 (Santa Cruz), APP (SIGMA), and β3-tublin (covance). The membrane was then washed in TBS/Tween 20 (0.05%), incubated for 1 hr with anti-mouse/rabbit horseradish peroxidase-conjugated antibodies (Jackson ImmunoResearch Laboratory), and washed three times in TBS/Tween 20. Proteins were visualized using immobilon-HRP (Millipore) or SuperSignal West Femto Kit (PIERCE) and ChemiDoc XRS (BIO-RAD).

### Brain volume

For computed tomography (CT) analysis of brain volume, mice were anesthetized with intraperitoneal injection of pentobarbital sodium, and then scanned using LaTheta experimental animal CT system (LCT-200, Aloka, Tokyo, Japan). Contiguous slice images were acquired and used for reconstruction of 3D images and for calculation of the interior content of the skull as the brain volume.

### Immunofluorescence

Immunostaining was performed as described previously^25^. Mice were deeply anesthetized with pentobarbital and transcardially perfused with 4% paraformaldehyde. Fixed tissues were cryoprotected by overnight immersion in 30% sucrose, followed by embedding in OCT compound. Sections of 14 μm thickness were attached to glass slides, whereas sections of 50 μm thickness were floated on PBS. For cultures of dissociated neurons, cells were fixed by incubation in 4% paraformaldehyde for 5 min at 37 °C.

For immunostaining, tissue sections and isolated cells were permeabilized with 0.1–0.5% Triton X-100 in PBS and incubated overnight with primary antibodies, including those recognizing cleaved caspase-3 (Pharmingen), NeuN (Millipore), GFAP (SIGMA), GFP (MBL), and ChAT (Millipore). Tissues and cells were then incubated with Alexa488- and/or Cy3-conjugated secondary antibodies and 1 µg/ml Hoechst 33342, followed by washing and mounting. Epifluorescent microscopy was carried out using an Axioimager A1 microscope (Carl Zeiss, Goettingen, Germany), BZ9000 and BZ-X710 (Keyence, Japan). Confocal microscopy was performed using an LSM510 microscope (Carl Zeiss).

### Activity monitoring

Activity monitoring was performed under 12: 12 h light: dark conditions or constant darkness as described previously^34^. 2.5 to 4 month-old animals were used for monitoring, and were allowed free access to food and water. All experiments were performed according to the guidelines for the care and use of laboratory animals of the human science department, Waseda University.

General locomotor activity was recorded with an infrared radiation sensor (F5B; Omron). Double-plotted actograms of locomotor activity are shown with 6 min epochs, and circadian rhythmicity of activity was analyzed by chi-square periodogram analysis with a p = 0.01 significant threshold line by CLOCKLAB software (Actimetrics). The free-running period of activity rhythm of the control and MKK7 cKO mice was calculated by the activity onset difference from day 15 to day 30.

### Gait analysis

Mice whose hindpaws were labeled with black ink were allowed to work on a runway (length: 40 cm. width: 4 cm, height: 10 cm), which was lined with fresh white paper for each run strip. Footprints were recorded in an unforced manner at least three times. The average distance of forward movement between each stride was measured as “gait length”. The average distance between left and right hind footprint was measured as “gait width”. Footprints made at the beginning and end of the run where the animal was initiating and finishing movement were excluded. Runs with interruption or hesitation such as wall climbing, grooming, and staying on the runway were not analyzed.

### Semi-thin sections of spinal cords

Spinal cords, which were fixed by perfusion with 2% glutaraldehyde-2% paraformaldehyde buffered with 0.1 M PB (pH 7.2) and further immersed in the same fixatives for 2 hours, were transversely sectioned into 1-mm blocks. Samples were postfixed with 2% Osmium tetroxide buffered with 0.1 M PB (pH 7.2), dehydrated with a graded series of alcohol, and embedded in Epon 812 (Taab Laboratory Equipment, Reading, UK). One-micrometer sections were cut with an ultramicrotome (UC6, Leica) and stained with toluidine blue.

### cDNA microarray

Total RNA was extracted from mouse brain using Trizol reagent (Invitrogen), and further purified using RNeasyR Mini Kit (Qiagen) as described previously^35^. The quality of RNA was initially assessed by electrophoresis on a 1.5% agarose gel, and further determined by absorption spectrophotometer (Agilent Bioanalyzer 2100 [Agilent, Palo Alto, CA]). cDNAs were synthesized by Low Input Quick Amp Labeling Kit. Cy3-Labeled cRNA was synthesized by *in vitro* transcription with T7 RNA Polymerase. Following fragmentation, 0.6 μg of cRNA was hybridized for 17 hours at 65 °C on the SurePrint G3 Mouse GE 8 × 60 K Microarray using Gene Expression Hybridization Kit. GeneChips were washed using the Gene Expression Wash Buffers Pack and scanned using Agilent DNA Microarray Scanner (G2565CA). Microarray data was processed using GeneChip Operating Software (Feature Extraction).

### Statistical analysis

Sample sizes were determined on the basis of pilot experiments and previous experience from similar experiments. To examine whether the data had the same variances, we analyzed them by F-test. As all the data were determined to be normally distributed, parametric statistics were used throughout. Data were analyzed by Student’s t-test or Welch’s t-test. All the t-tests were performed as two-tailed t-tests. The statistical test used for each experiment is stated in the figure legend.

## Results

### The JNK pathway is constitutively activated in the mouse adult brain

**Figure 1:**
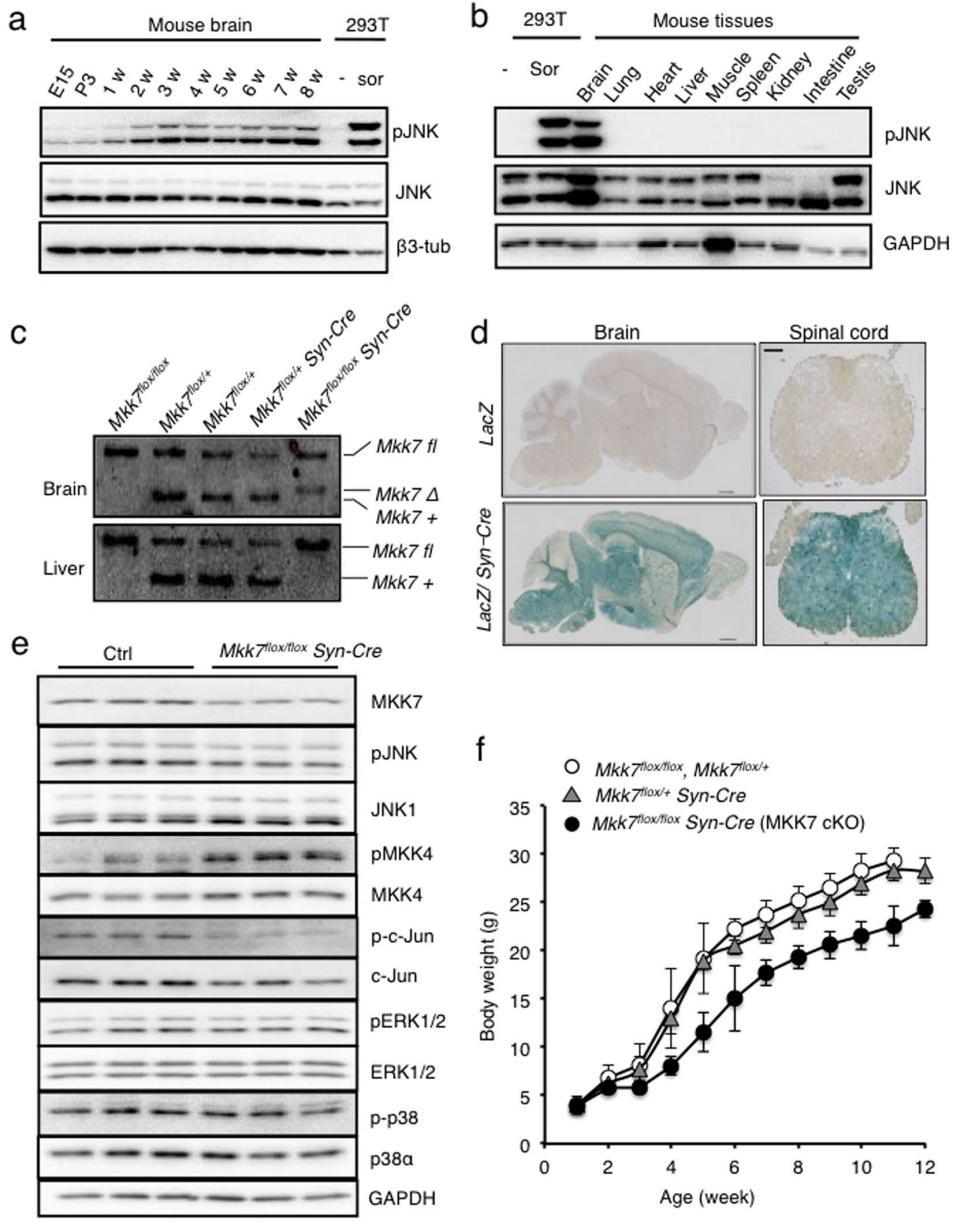
Generation of neuron-specific MKK7 conditional knockout mice. (**a**) Immunoblot analysis of phospho-JNK in the mouse brain from embryonic to adult stages. Sor indicates sorbitol treatment as a positive control of JNK activation. β3-tublin (β3-tub) is the loading control. (**b**) Immunoblot analysis of phospho-JNK in adult mouse tissues. Extracts of tissues from WT mice were normalized by protein concentration. (**c**) Southern blotting analysis of *Mkk7* gene deletion. HindIII-restricted genomic DNA was prepared from 3 week-old mice of the indicated genotypes. (**d**) Analysis of regions expressing Syn-Cre by β-gal staining. Brain and lumber spinal cord were prepared from 4 month-old LacZ reporter mice and Syn-Cre/LacZ double transgenic mice. Brain: Scale bar indicates 1 mm; Spinal cord: Scale bar indicates 300 μm. **(e)** Analysis of MAPKs activation in brain. Extracts of brain from 3 month-old MKK7 cKO and control mice were prepared and immunoblotted to detect MKK7, MKK4, JNK, ERK, p38 and c-Jun activities. GAPDH is the loading control. (**f**) Body weight of MKK7 cKO mice and control mice from 1–12 week-old. *Mkk7* ^*flox/flox*^ *, Mkk7* ^*flox/+*^, *Mkk7* ^*flox/+*^ *Syn-Cre* and *Mkk7* ^*flox/flox*^ *Syn-Cre*: n > 3.

To examine the activation of JNK signaling over the lifetime of the mouse, we performed a time-course analysis of JNK phosphorylation in wild-type mouse brain. We found constitutive activation of JNK through embryonic to adult stages in mice brain (Fig. 1a). This constitutive JNK activation was not observed in other tissues including lung, heart, liver, muscle, spleen, kidney, intestine and testis (Fig. 1b). Phosphorylation of the JNK substrate c-Jun was also constitutively upregulated in brain, but not in other organs (Fig. S2). These results suggest potential important roles of JNK signaling in the adult brain.

To investigate the physiological roles of JNK signaling specifically in the adult brain, we generated conditional MKK7 knockout mice by using *Synapsin1-Cre (Syn-Cre)* transgenic mice in which *Cre* recombinase is expressed in neurons but not glial cells^33, 36^. The deletion band was detected only in *Mkk7* ^*flox/flox*^ *Syn-Cre* genomes from the brain but not other organs such as liver by Southern blotting (Fig. 1c). *Mkk7* ^*flox/flox*^ *Syn-Cre* mice were born at Mendelian ratios. To confirm the deleted patterns by *Syn-Cre*, we used LacZ reporter mice. Deletion by Syn-Cre was detected in most regions of the brain and spinal cord as previously reported (Fig. 1d)^36^. To examine MKK7 expression in *Mkk7* ^*flox/flox*^ *Syn-Cre* (MKK7 cKO), we performed immunoblotting and found decreased MKK7 expression (less than 60%) (Fig. 1e). MKK7 expression was not totally eliminated, presumably because MKK7 expression remained in glial cells. JNK phosphorylation was also reduced (50%) in MKK7 cKO brain. In contrast, activation of MKK4 was increased in MKK7 cKO mice brain, consistent with a previous report^25^. Activation of other MAPKs such as ERK1/2 and P38 were not altered in MKK7 cKO brain. MKK7 cKO mice were indistinguishable from their control littermates at birth (Fig. S3). However, their growth was slower compared to control mice (around 80%), but essentially progressed as normal (Fig. 1f).

### Neuron-specific *Mkk7* deletion leads to enlarged brain

**Figure 2:**
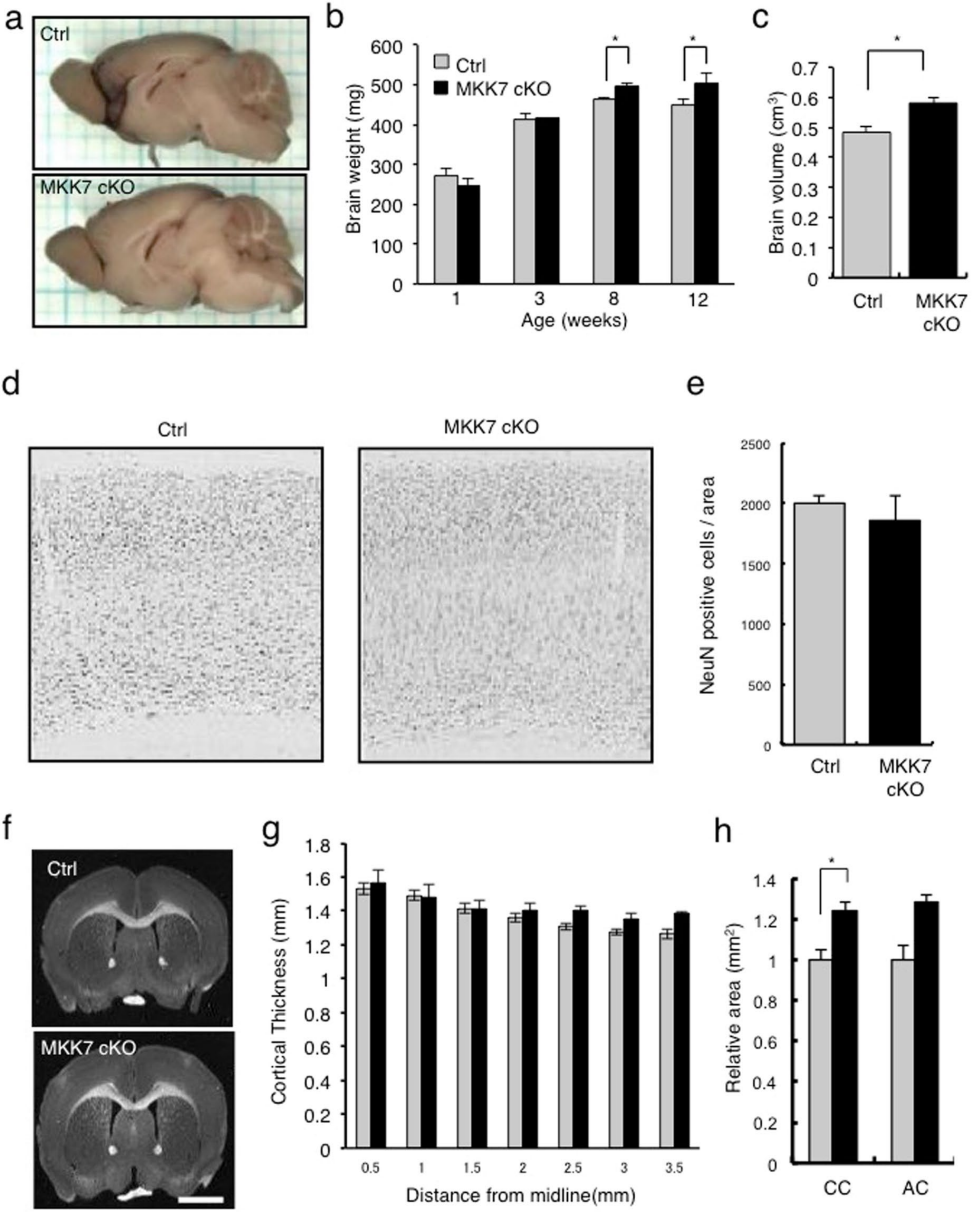
Analysis of brain size. (**a**) Gross appearance of control and MKK7 cKO mouse brain at 4 months-old. (**b**) Brain wet weights of control and MKK7 cKO mice between 1–12 weeks-old. (**c**) Brain volumes of 8 month-old control and MKK7 cKO mice. Volumes were calculated from contiguous slice images acquired by CT scan. (**d**) Immunostaining of cortical neurons. Sections were prepared from 3 month-old control and MKK7 cKO mice and stained with anti-NeuN antibody. (**e**) Numbers of NeuN positive cells per section. (**f-h**) Coronal sections were prepared from 3 month-old control and MKK7 cKO brains. (**g**) Thickness of cortex. (**h**) Relative areas of white matter. CC: Corpus Callosum, AC: Anterior commissure. *p < 0.05.

To examine brain size, we measured brain weight of control and MKK7 cKO mice. We found that brains of MKK7 cKO mice were heavier than controls from 2 months of age (Fig. 2a, b). CT scan analysis revealed that brain volumes of MKK7 cKO mice were also larger than controls (Fig. 2c). To identify the mechanism of this macrocephaly, we first investigated the possibility of increased cell numbers by immunostaining neurons with a NeuN antibody and counting the number of neurons in the cortex. There was no significant difference in neuronal number between control and MKK7 cKO mice (Fig. 2d, e). The cortical thickness of MKK7 cKO brains was also not different from that of the control (Fig. 2f and g). However, we did find that white matter area, specifically the corpus callosum (CC), was significantly increased in MKK7 cKO mice (Fig. 2f and h). The anterior commissure (AC) was also increased in MKK7 cKO mice. Dendritic arborization was also examined using *Mkk7* deleted primary culture neurons that were sparsely labeled with GFP. We performed Sholl analysis and found that the dendrites of *Mkk7* deleted neurons showed higher complexity (i.e., increased numbers of intersections) than control neurons (Fig. S4). These results suggest that increased arborization and/or number of neurites cause the enlarged brains in MKK7 cKO mice.

### Neuron-specific *Mkk7* deletion leads to impaired behavioral rhythms and decreased activity

**Figure 3:**
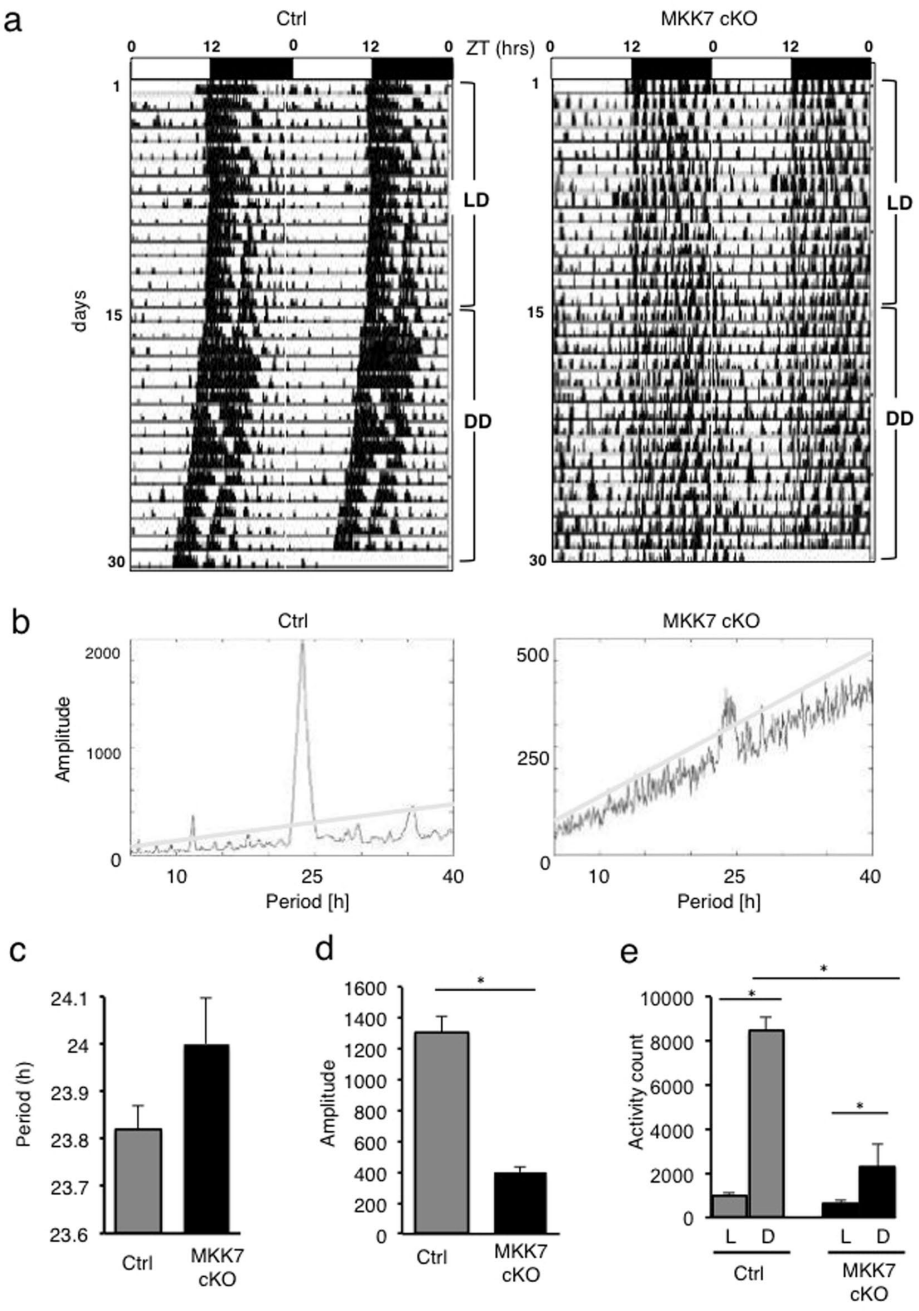
Analysis of behavioral patterns. **(a)** Representative double-plotted actograms of locomotor activity in MKK7 cKO and control mice at 2.5 to 4 months. Zeitgeber time (ZT) 0 corresponds to the time point of lights-on and ZT12 is the lights-off time point. (**b**) χ^2^ periodograms analyzed during DD conditions. (**c**) Averaged circadian periods of behavioral rhythms under DD condition. (**d**) Averaged periodogram amplitude of behavioral rhythms under DD condition. (**e**) Averaged daily activity count of MKK7 cKO and control mice under LD conditions. Control: n = 10, MKK7 cKO: n = 6. *p < 0.05.

To elucidate whether the role of MKK7 in circadian regulation also occurs at the animal level, we monitored the free running locomotor activity of MKK7 cKO mice in home cages with infrared area sensors, and activity records under light and dark (LD) conditions followed by a constant dark (DD) condition were depicted as actograms with 2 cycles plotted next to each other (double-plot) (Fig. 3a). In LD conditions, as expected, the mice showed 24 hrs periods in their behavioral rhythms because their activities were regulated by the light^37^. While, in the DD condition, activity becomes regulated by their internal circadian clock: control mice displayed 23.8 ± 0.2 hrs periods, however, MKK7 cKO mice displayed slightly longer periods of circadian behavioral rhythms (24 ± 0.2 hrs) (Fig. 3b, c). Importantly, we also found that specifically the amplitude of the behavioral rhythms was significantly reduced in MKK7 cKO mice (Fig. 3d). In addition, the average activity counts under LD conditions were significantly reduced in MKK7 cKO mice compared to control mice (Fig. 3e). Taken together, these results indicate that *Mkk7* deletion impairs circadian rhythms and reduces animal activity.

### Neuron-specific *Mkk7* deletion leads to progressive motor dysfunctions

**Figure 4:**
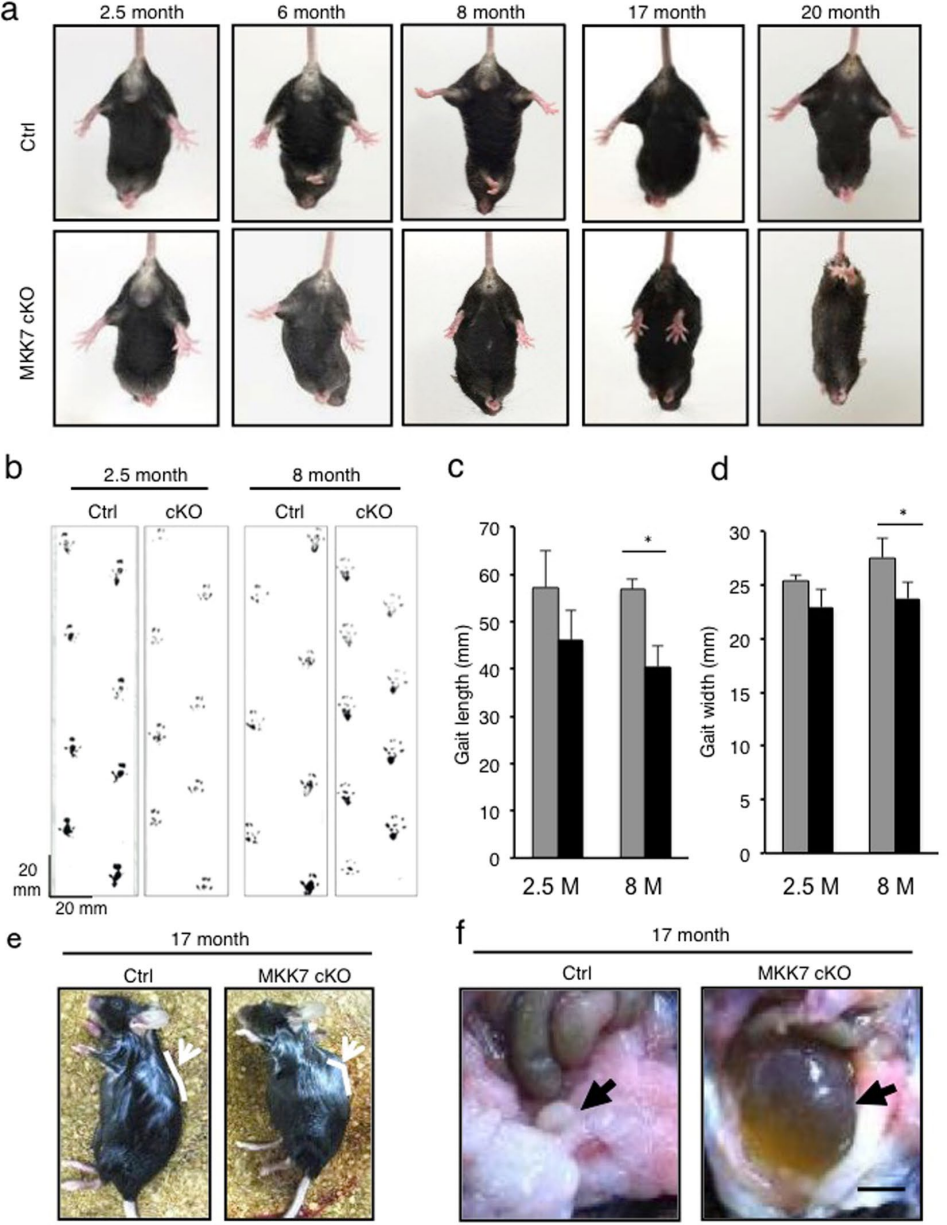
Analysis of motor ability. (**a**) Angles of hind-limb extinction when lifted by the tail at the indicated age. (**b**) Representative footprint patterns of MKK7 cKO and control mice at the indicated age. (**c**) Average gait length (distance of forward movement of each stride). (**d**) Average gait width (distance between left and right of footprint). 2.5 months Control: n = 3; 2.5 months MKK7 cKO: n = 4; 8 months Ctrl: n = 4; 8 months MKK7 cKO: n = 5. *p < 0.05 (**e**) Posture of 17 month-old MKK7 cKO and control mice. (**f**) Bladder of 17 month-old MKK7 cKO and control mice. Scale bar, 5 mm.

**Figure 5:**
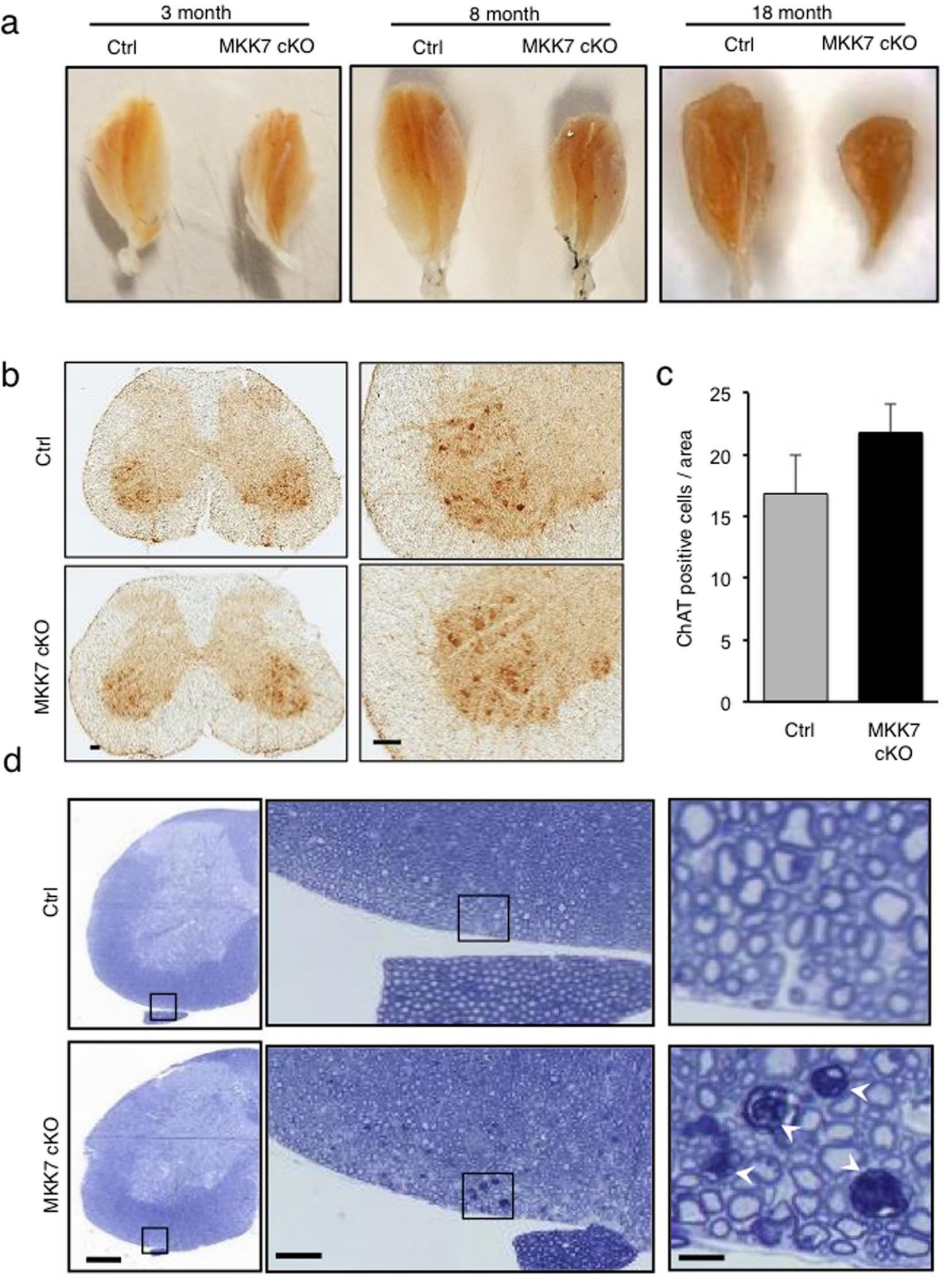
Analysis of muscle and spinal cord. **(a**) Dissected gastrocnemius muscles from MKK7 cKO and control mice at the indicated ages. **(b**) Immunostaining of ChAT positive motor neurons in spinal cord. LSC were collected from 9 month-old MKK7 cKO and control mice. Scale bar, 100 μm. (**c**) Averaged numbers of ChAT positive cells per section. Ctrl: n = 4; MKK7 cKO: n = 5. (**d**) Toluidine blue staining of semi-thin sections of LSC. LSC were collected from 9 month-old MKK7 cKO and control mice. Arrowheads indicate degenerating axons. Left panels, scale bar indicates 250 μm. Middle panels are areas magnified from the left panels. Scale bar, 50 μm. Right panels are magnified from the middle panels. Scale bar, 10 μm.

To examine motor ability, we analyzed the hind-limb reflex under tail suspension. The angle of the hind-limb was indistinguishable between control and MKK7 cKO mice until 6 month-old (Fig. 4a). At 8 months, the hind-limb reflex gradually decreased in MKK7 cKO mice. MKK7 cKO mice finally became unable to extend their hind-limb at 20 months of age. The tail posture of MKK7 cKO mice also decreased compared to control mice at 8 months, and MKK7 cKO mice attached both their tails and bodies to the floor at 20 months (Fig. S5). Footprint analysis revealed that both gait length and width were also significantly shortened in 8 month-old MKK7 cKO mice (Fig. 4b-d). MKK7 cKO mice displayed gait abnormality and paralysis especially in their hind-limb at 20 months (Supplemental movie 1–4). In addition, MKK7 cKO mice displayed kyphosis and urinary retention at 17 months (Fig. 4e, f). To analyse the effect on the musculature, we dissected skeletal muscle of the hind-limb. MKK7 cKO showed progressive and severe atrophy of the gastrocnemius muscle at 18 months (Fig. 5a). To determine the pathological basis for this progressive muscle atrophy, we analysed neuronal degeneration in the spinal cord. Choline acetyl-transferase (ChAT) staining, which specifically stains motor neurons, revealed that motor neuron levels were not reduced in the lumber spinal cord (LSC) of MKK7 cKO mice at 9 months (Fig. 5b, c). Next, we investigated axonal changes by toluidine blue staining of semi-thin cross sections embedded in epoxy resin. We found abnormal (degenerating) axons in the ventral area of the LSC of MKK7 cKO mice at 9 months (Fig. 5d). Thus, MKK7 deletion in differentiated neurons leads to axonal neuropathy at 9 months and muscle atrophy at 18 months, which likely cause the severe gait abnormality observed at 20 months.

### Neuron-specific *Mkk7* deletion leads to accumulation of APP and JIP1

**Figure 6:**
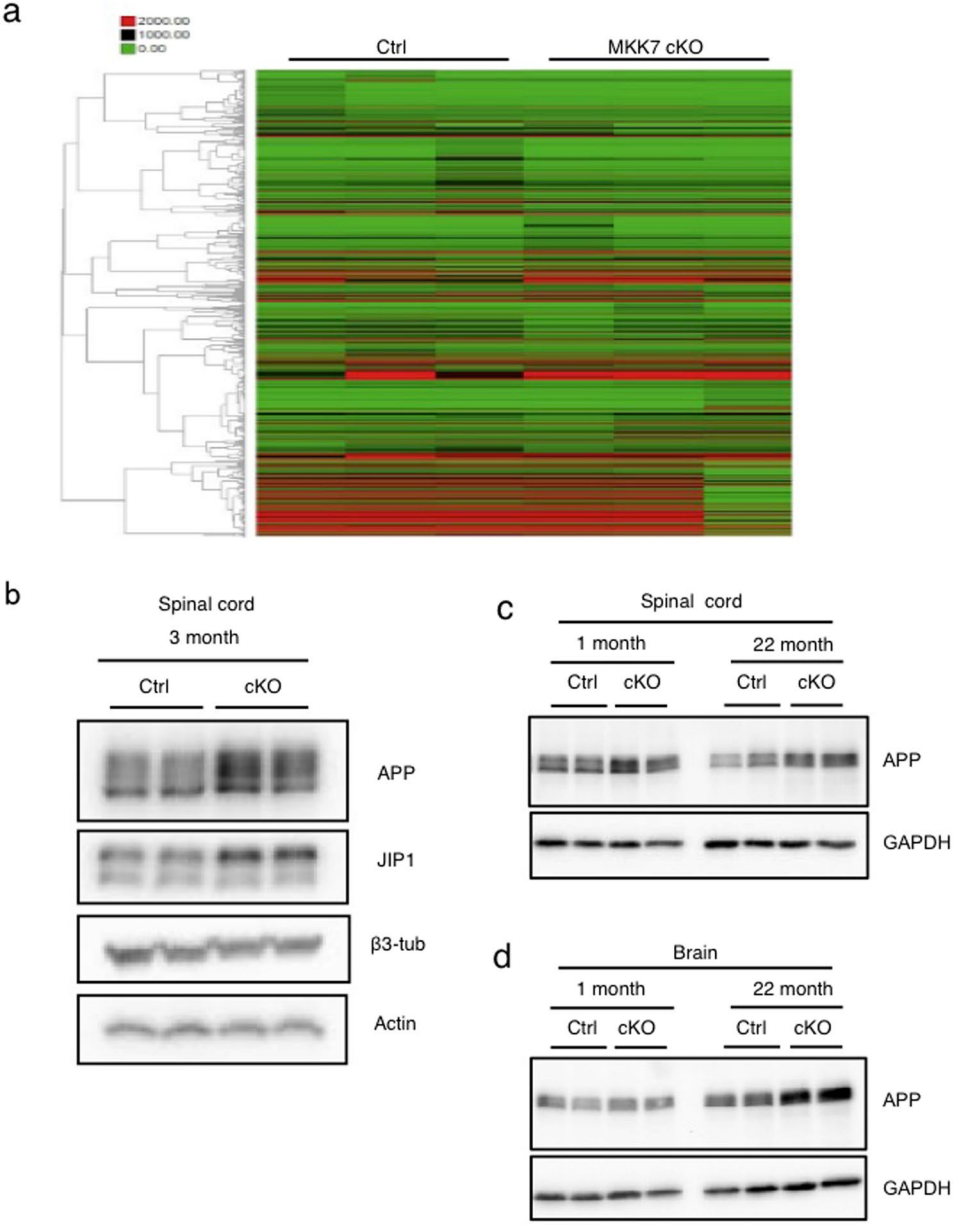
Analysis of APP and JIP1 protein levels in nervous tissues. (**a**) cDNA microarray and hierarchical cluster analysis of MKK7 cKO mice brain. Heat map showing expression patterns of 8 month-old MKK7 cKO and control brain. (**b**) Immunoblotting of APP and JIP1 in spinal cord. Extracts of spinal cord from 3 month-old MKK7 cKO and control mice were used. β3-tublin and actin are loading controls. (**c** and **d**) Immunoblotting of APP both in the spinal cord and brain. Extracts of brain and spinal cord were prepared from MKK7 cKO and control mice at the indicated ages. GAPDH is the loading control.

To identify the molecular mechanism for the motor deficit, we first analyzed gene expression by cDNA microarray of MKK7 cKO brain at 8 months, when the motor defects were observed. Unexpectedly, the expression levels of most genes were unchanged between the MKK7 cKO and control mice in a genotype-dependent manner (Fig. 6a). MKK7-JNK signaling also activates downstream effector proteins directly rather than by regulating gene expression.

To examine the phosphorylation status of the JNK substrates, we performed two-dimensional (2D) gel analysis using an anti-phospho-threonine-proline (pTP) antibody. The TP sequence is one of JNKs phosphorylation site consensus sequences. Several spots of pTP signal decreased in 3 month-old MKK7 cKO brain compared to wild-type brain (Fig. S6), suggesting that MKK7-JNK phosphorylates several target proteins in the adult brain. Further, we analyzed MKK7-JNK target proteins involved in neural function. We found accumulation of amyloid-beta precursor protein (APP) and JIP1 in MKK7 cKO spinal cords (Fig. 6b). APP accumulation was also observed in the brain of MKK7 cKO mice at 22 months (Fig. 6c and d). These results indicate that the metabolism of proteins involved in neural function are impaired in MKK7 cKO mice.

## Discussion

Previously, it was reported that stress-induced JNK activation generates a proapoptotic signal in the brain^15–18^. In this study, we found constitutive activation of JNK in the brain (Fig. 1a and b), but this activation may not cause apoptosis. This discrepancy may be due to differences in the distribution of JNKs and it’s target substrates between different cellular conditions. It has been reported that JNKs translocate to the nucleus and mitochondria and modulate proapoptotic factors such as the Bcl-2 family under stress conditions^38–41^. Several lines of evidence have shown that JNKs locate in neurites and phosphorylate cytoskeletal targets such as MAP1b, MAP2, DCX and SCG10 without stress stimuli^11, 42–45^. Further analysis of temporal and spatial JNK activation patterns at the single cell level in neurons is needed.

Activity monitoring revealed that MKK7 cKO mice showed impaired behavioral rhythms including elongated period length (Fig. 3a-d). Our previous report provided evidence that one of the clock proteins PER2 was phosphorylated and stabilized by the MKK7-JNK signal^31^. In addition, Yoshitane *et al*. reported that genetic knockout of JNK3 resulted in a longer free-running period^19^. They also identified BMAL1 as a JNK target *in vitro*, and BMAL1 phosphorylation was also decreased in the suprachiasmatic nucleus (SCN) of JNK3 KO mice, where the master clock is located in mammals^46^. These findings suggest that MKK7-JNK signaling phosphorylates clock proteins and controls the period length of its transcription loops in SCN neurons and behavioral rhythm at the animal level.

In this study, MKK7 cKO mice showed age-dependent motor dysfunctions caused by peripheral axonal neuropathy after 8 months (Fig. 4 and Fig. 5). Previously, it was reported that loss of JNK1 or MKK4 caused motor phenotypes: *Jnk1* ^*−/−*^ mice showed impairment of fine motor coordination and balance accompanied by abnormal dendritic architecture in the motor cortex^47^; *MKK4* ^*flox/flox*^ *Nestin-Cre* caused ataxia and awkward gait at P15-P16 due to misalignment of Purkinje cells in the cerebellum^24^. These motor phenotypes could be caused by secondary effects of impaired neural development. Thus, our finding is the first report that MKK7-JNK signaling is essential for maintenance of neural function and motor ability in aged mice.

Phenotypes of MKK7 cKO mice such as age-dependent motor deficits, abnormal axons and JIP1/APP protein accumulation (Figs. 5 and 6) are similar to those observed in mice lacking KIF5A, which is a kinesin-1 motor protein^48^. KIF5a knockout mice also showed age-dependent motor deficits (hind-limb paralysis), axonal degeneration and protein accumulation due to impaired axonal transport. JNKs have been shown to regulate axonal transport: JNK3 directly phosphorylates motor protein kinesin-1 and induces its dissociation from microtubules^49^; JIP1 mutants lacking the JNK phosphorylation site altered the directionality of APP transport in axons^50^. Therefore, perturbation of axonal transport may cause the accumulation of transport cargoes and adaptor proteins including APP and JIP1. Although accumulation of APP itself may not directly cause neuronal degeneration, transport of several important molecules such as synaptic proteins, mitochondria and neurofilaments are affected by JNK^51^. Thus, dysregulation of axonal transport might underlie the axonal neuropathy and severe motor deficit observed in MKK7 cKO mice in an age-dependent manner.

Recently, human genetics studies identified an involvement of the MKK7-JNK pathway in psychiatric disorders such as autism spectrum disorders and schizophrenia^52, 53^. In addition, mice haploinsufficient for MKK7 also displayed impaired attention and cognitive processing^54^. Our results also show that MKK7 inactivation leads to abnormalities in circadian rhythm and behavioral activity, which have also been associated with psychiatric disorders (Fig. 3e)^55, 56^. Thus, a reduction of the MKK7-JNK signal may underlie some of the neurochemical and behavioral changes in psychiatric disorders and may be a novel candidate target for developing new treatments.

## Electronic supplementary material

Supplementary Info

Video 1

Video 2

Video 3

Video 4
