# Supplementary material for: Age-dependent motor dysfunction due to neuron-specific disruption of stress-activated protein kinase MKK7
Source: Sci Rep. 2017 Aug 4;7:7348. doi: 10.1038/s41598-017-07845-x (PMC5544763; doi:10.1038/s41598-017-07845-x)
Supplement: Supplementary file 1 — Supplementary Info [file 41598_2017_7845_MOESM1_ESM.pdf]

Supplementary Information

Age-dependent motor dysfunction due to neuron-specific disruption of stress-activated protein kinase MKK7

Tokiwa Yamasaki[1]*, Norie Deki-Arima[1]*, Asahito Kaneko[2], Norio Miyamura[1], Mamiko Iwatsuki[3], Masato Matsuoka[3], Noriko Fujimori-Tonou[4], Yoshimi Okamoto-Uchida[1], Jun Hirayama[1], Jamey D. Marth[5], Yuji Yamanashi[6], Hiroshi Kawasaki[7], Koji Yamanaka[8], Josef M. Penninger[9], Shigenobu Shibata[2] and Hiroshi Nishina[1]

[1] Department of Developmental and Regenerative Biology, Medical Research Institute, Tokyo Medical and Dental University (TMDU), 1-5-45 Yushima, Bunkyo-ku, Tokyo 113-8510, Japan

[2] Laboratory of Physiology and Pharmacology, School of Advanced Science and Engineering, Waseda University, Tokyo, Japan.

[3]Department of Hygiene and Public Health I, Tokyo Women's Medical University, Tokyo, Japan.

[4]Laboratory for Molecular Dynamics of Mental Disorders, RIKEN Brain Science Institute, Wako, Saitama, 3510198, Japan.

[5]Center for Nanomedicine, SBP Medical Discovery Institute, Department of Molecular, Cellular, and Developmental Biology, University of California Santa Barbara, Santa Barbara, California, USA.

[6]Division of Genetics, Department of Cancer Biology, The Institute of Medical Science, The University of Tokyo, Shirokanedai, Minato-ku, Tokyo, 108-8639, Japan.

[7]Department of Medical Neuroscience, Graduate School of Medical Sciences; Brain/Liver Interface Medicine Research Center, Kanazawa University, Kanazawa, Japan.

[8]Department of Neuroscience and Pathobiology, Research Institute of Environmental Medicine, Nagoya University, Nagoya, Japan.

[9]IMBA, Institute of Molecular Biotechnology of the Austrian Academy of Sciences, Vienna, Austria

*These authors contributed equally to this work.

Correspondence and requests for materials should be addressed to H.N. (e-mail: nishina.dbio@mri.tmd.ac.jp)

Supplementary Methods

Preparation of cortical neurons and Sholl analysis

In utero electroporation and cortical neurons culture were performed as described previously[1] with slight modifications. Briefly, pregnant mice were anesthetized with sodium pentobarbital and the uterine horns were exposed. Approximately 1-2 μl of pCAG-GFP plasmid DNA solution (1-3 mg/ml) was injected into the lateral ventricle of each embryonic brain using a pulled glass micropipette. Each embryo within its uterus was then placed between tweezer-type electrodes with a diameter of 5 mm (CUY650-P5; NEPA Gene, Chiba, Japan). Square electric pulses (45 V, 50 ms) were passed five times at 1 s intervals using an electroporator (ECM830, BTX). Care was taken to quickly place embryos back into the abdominal cavity to avoid excessive temperature loss. The wall and skin of the abdominal cavity were sutured, and embryos were allowed to develop normally.

At E16.5, cortices were dissected from control or *Mkk7*$^{flox/flox}$ *Nestin-Cre* embryos that had been electroporated at E15.5. The cortices were dissected, treated with papain, and plated onto poly-L-lysine-coated coverslips, and maintained in Neurobasal medium (Invitrogen). Cortical neuron cultures were maintained in 5 % CO_2 at 37 ºC. After 6 days *in vitro*, we fixed cells and counted the numbers of intersections with dendrite and concentric circles centered at the cell body.

Comprehensive analysis of phosphorylated proteins

Phosphorylated proteins were purified from mouse brain lysate by column chromatography of Phos-Tag® agarose (Wako). The 2D-PAGE was performed as previously described with slight modifications[2]. Extracts (150 μg) were subjected to 2D-PAGE using an immobilised pH gradient for first-dimension isoelectric focusing (pH4-7) (IEF) (ZOOM IPGRunner System; Invitrogen) according to the manufacturer's instructions. Phosphorylated proteins were detected by immunoblotting of anti-phospho-Thr-Pro (Cell Signaling).

Supplementary Figures

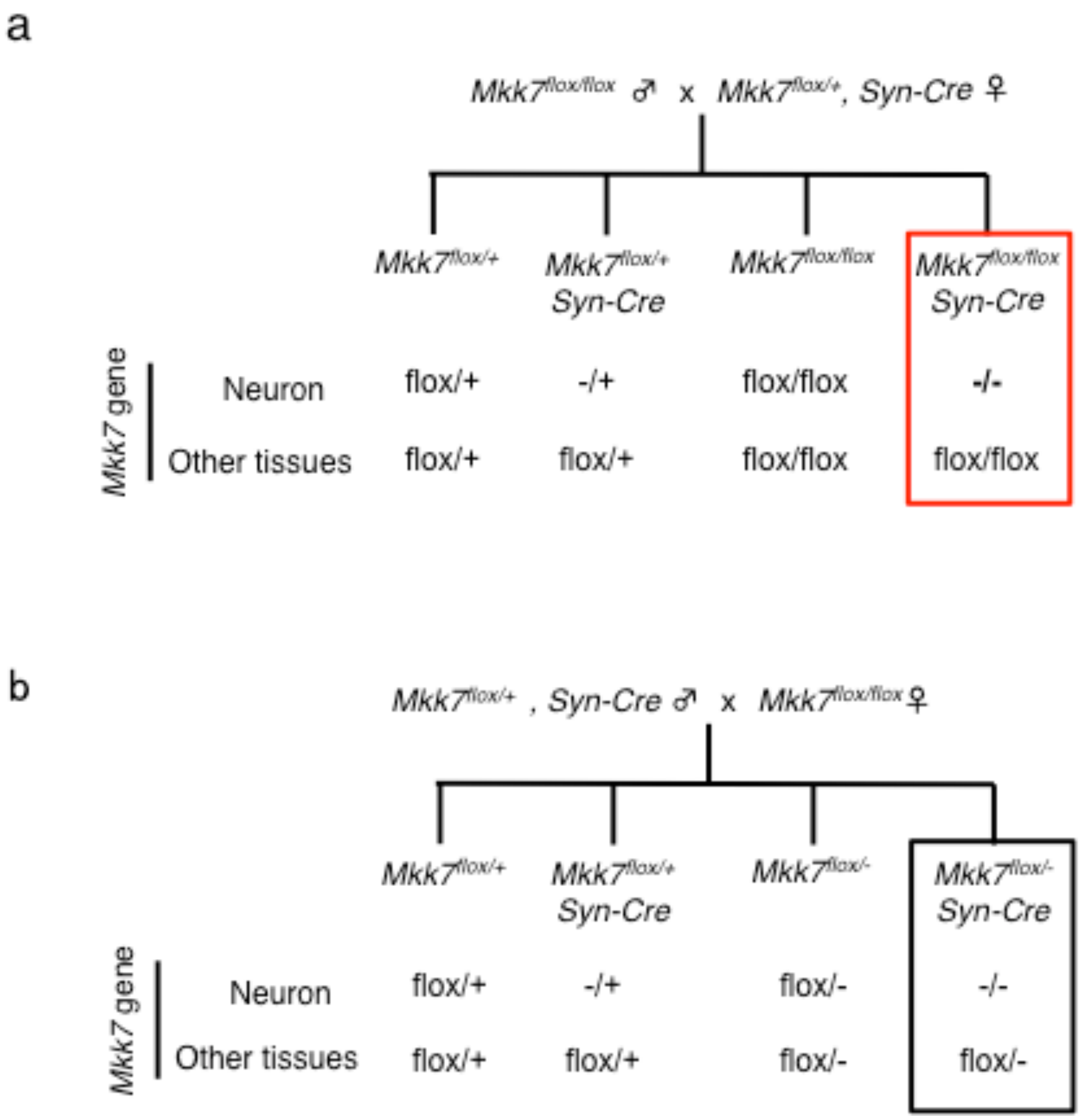

Supplementary Figure S1: Breeding scheme for generating MKK7 cKO mice and control animals

(**a**) Appropriate breeding scheme to obtain neuron specific MKK7 cKO mice. (**b**) Inappropriate breeding scheme.

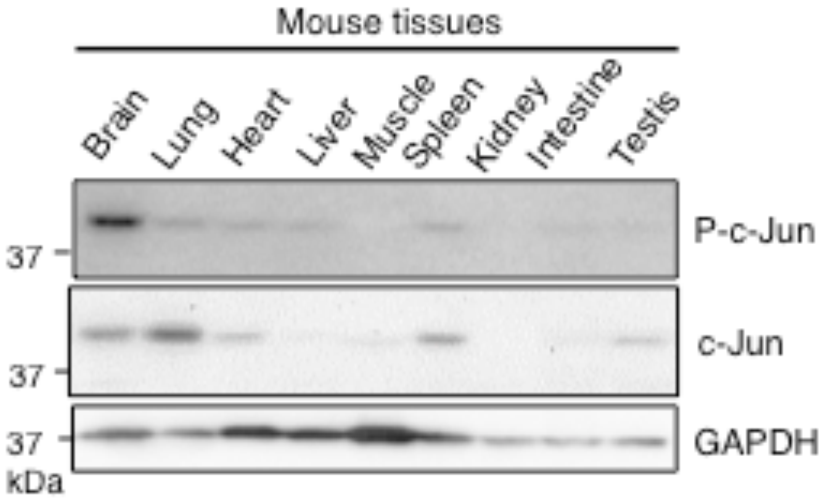

Supplementary Figure S2: Immunoblot analysis of phospho-c-Jun in adult mouse tissues.

Extracts of tissues from WT mice were normalized by protein concentration.

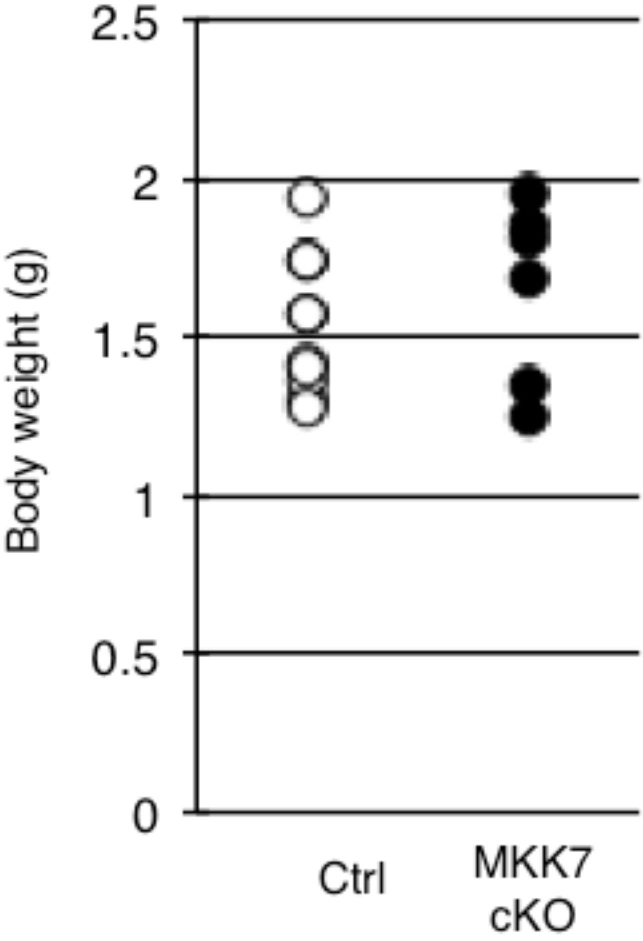

Supplementary Figure S3: Analysis of body weights at birth

Body weights of newborn mice. White circle: control mice, Black circle: MKK7 cKO mice.

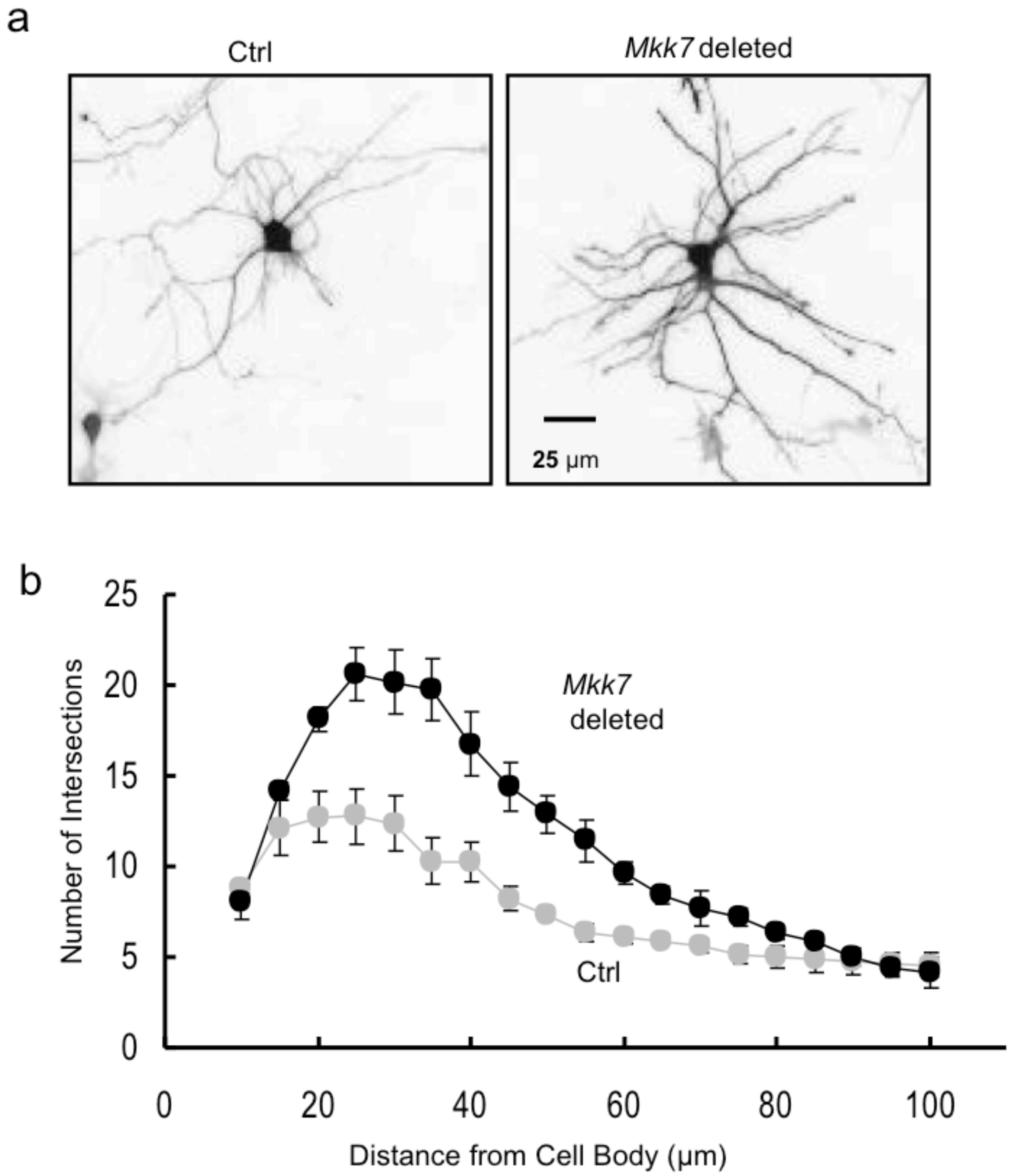

Supplementary Figure S4: Analysis of dendritic morphology in *Mkk7* deleted neurons

(**a**) Representative images of dendrites of primary cultured cortical neurons. *Mkk7* deleted neurons were labeled by GFP. (**b**) Sholl analysis of *Mkk7* deleted neurons versus control neurons.

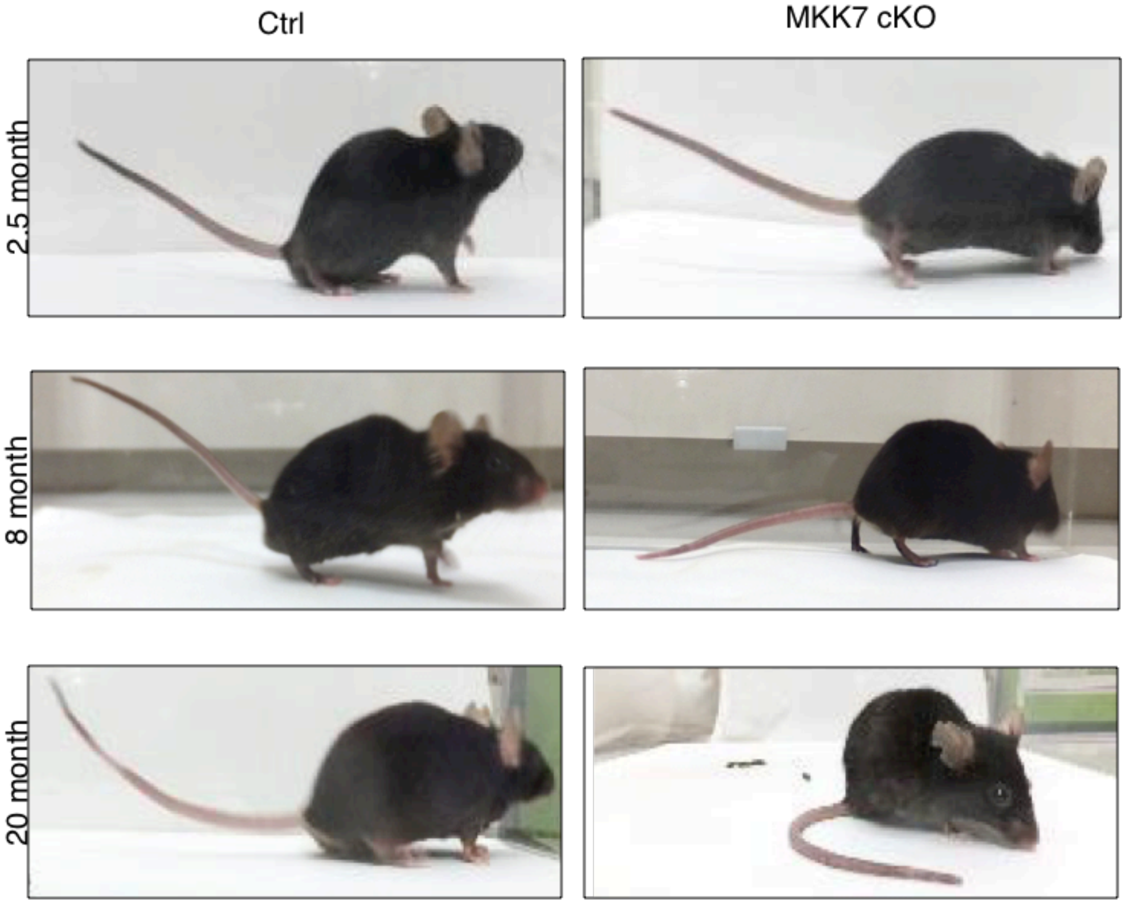

Supplementary Figure S5: Observation of tail posture

Photographs of tails of MKK7 cKO and control mice at 2.5, 8 and 20 month-old.

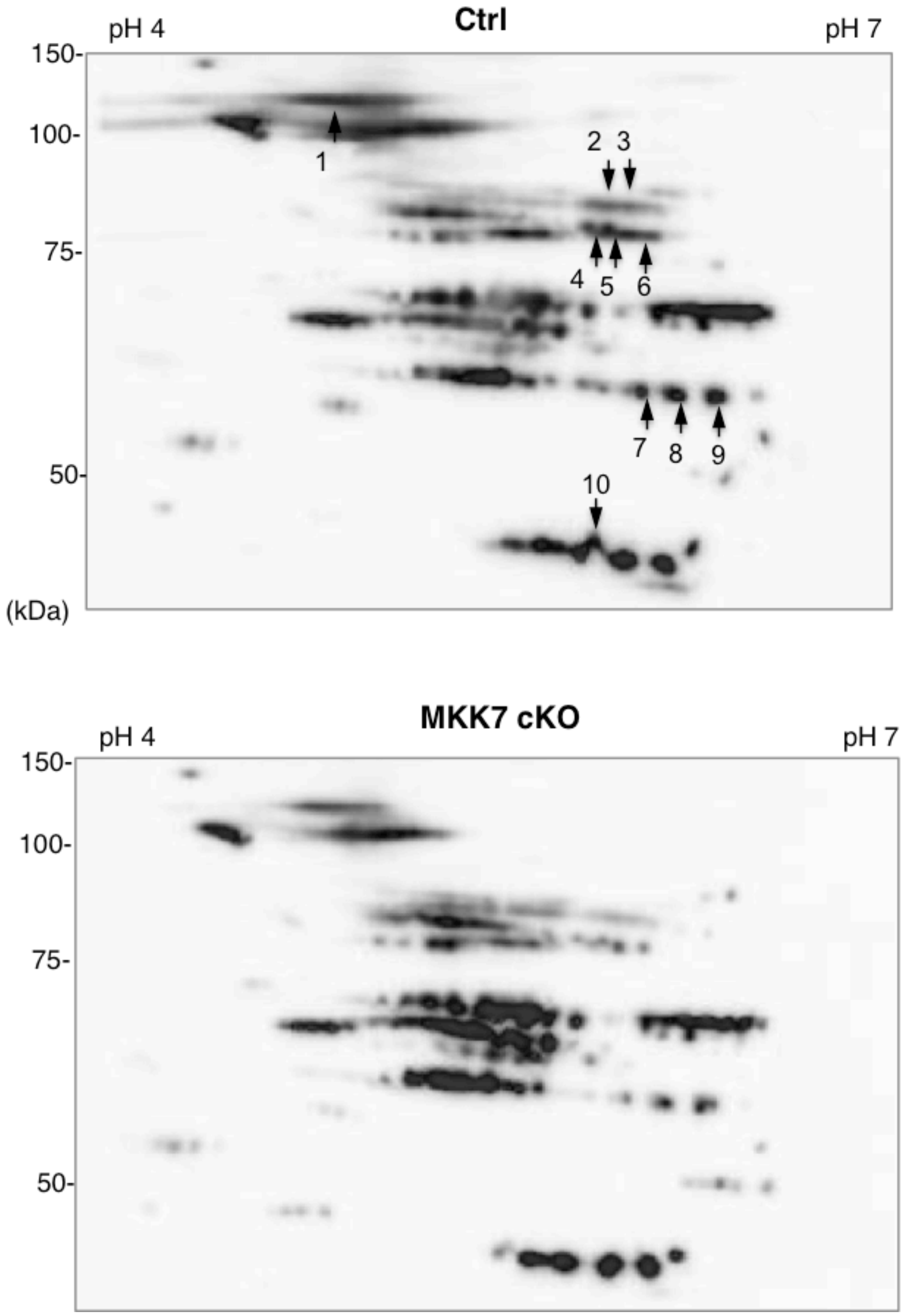

Supplementary Figure S6: Comprehensive analysis for JNK substrate in adult brain

Immunoblotting for Phospho-Thr-Pro after 2D-PAGE. Extracts were prepared from 3 month-old MKK7 cKO and control brain.

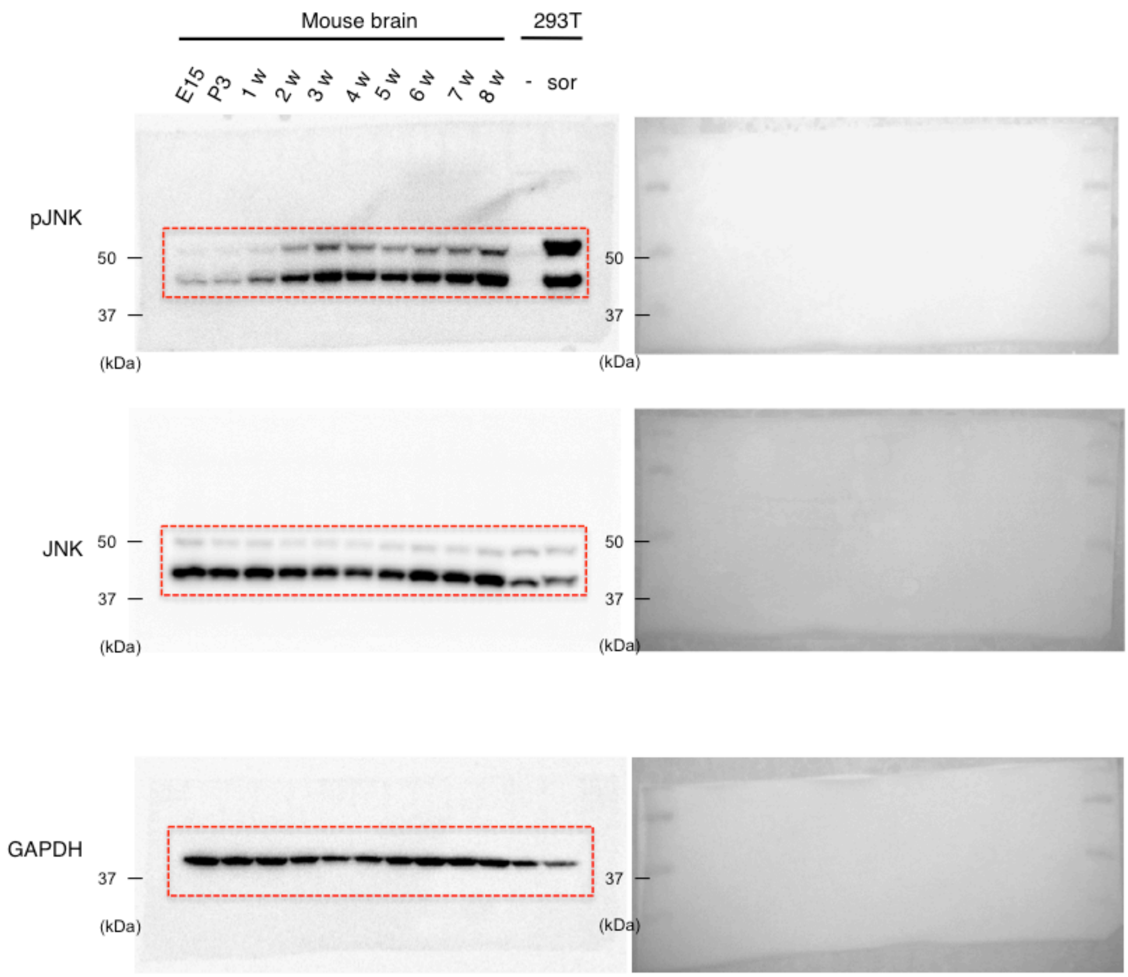

Supplementary Figure S7: Unprocessed scanned images of Figure 1a.

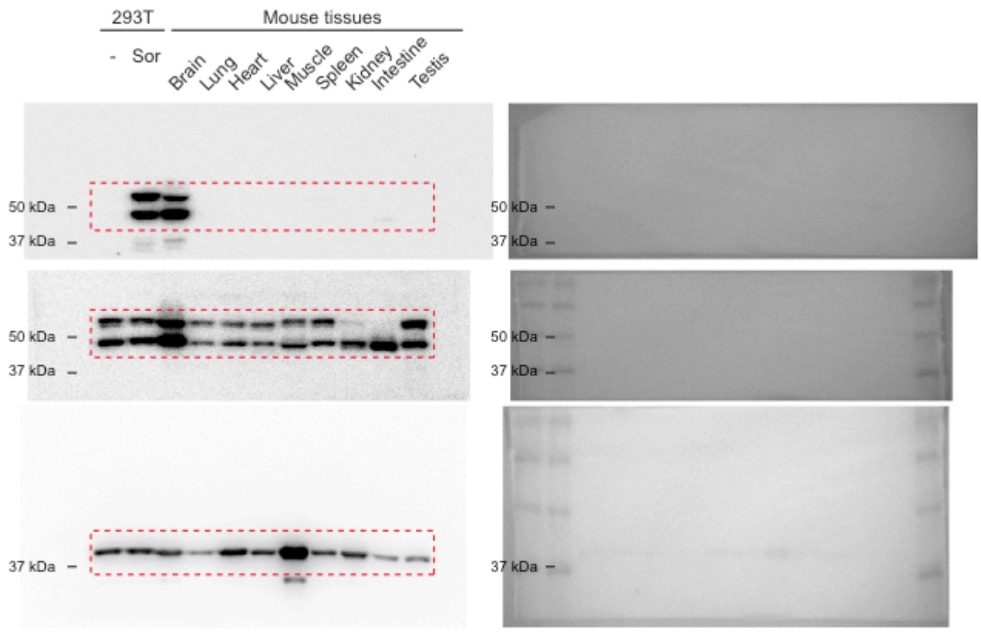

Supplementary Figure S8: Unprocessed scanned images of Figure 1b.

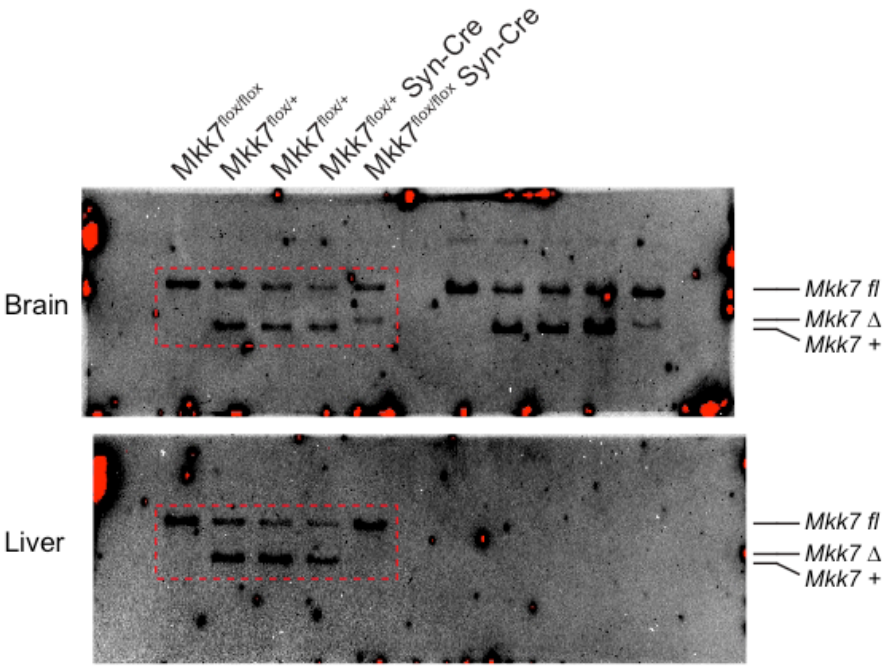

Supplementary Figure S9: Unprocessed scanned images of Figure 1c.

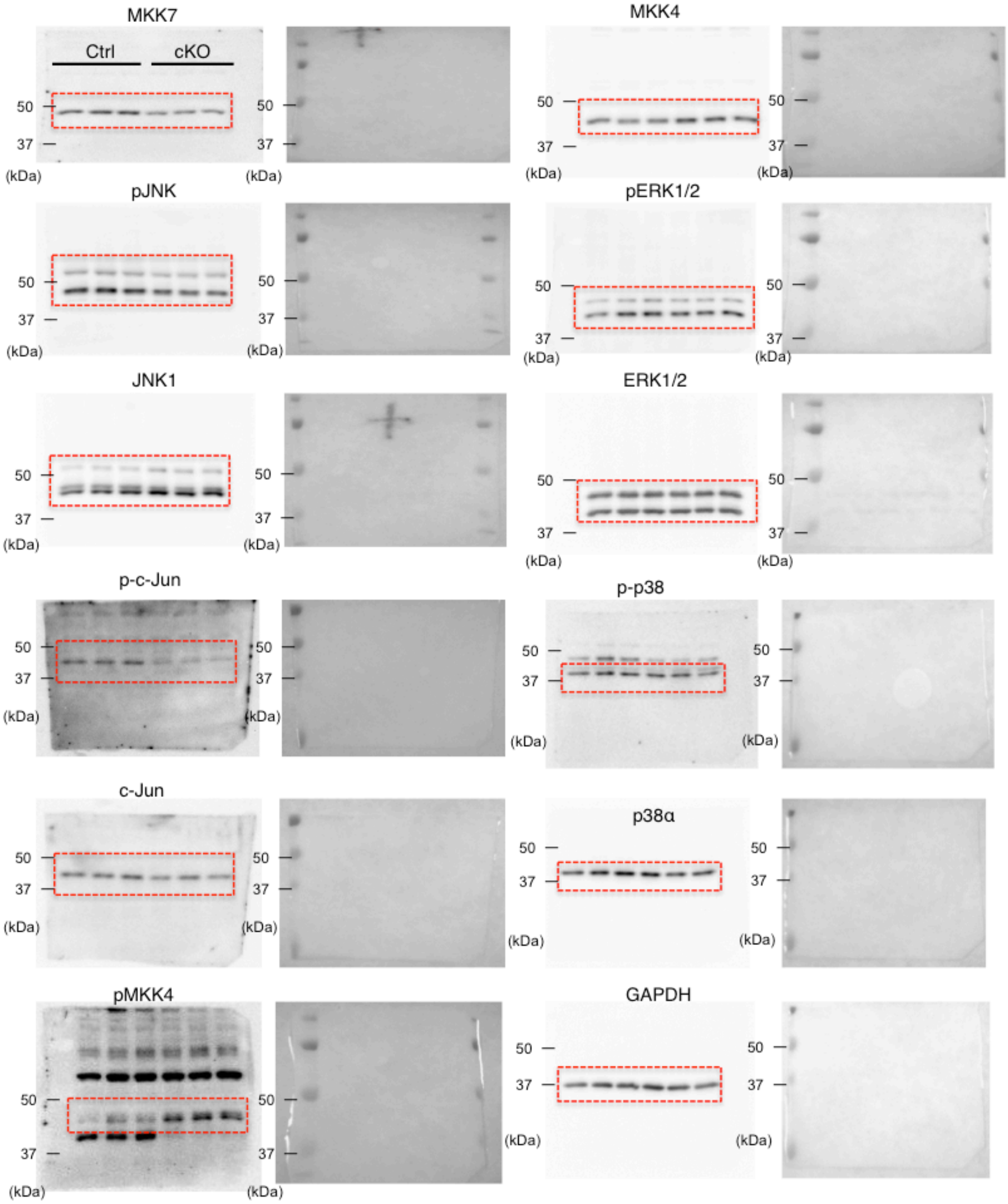

Supplementary Figure S10: Unprocessed scanned images of Figure 1e.

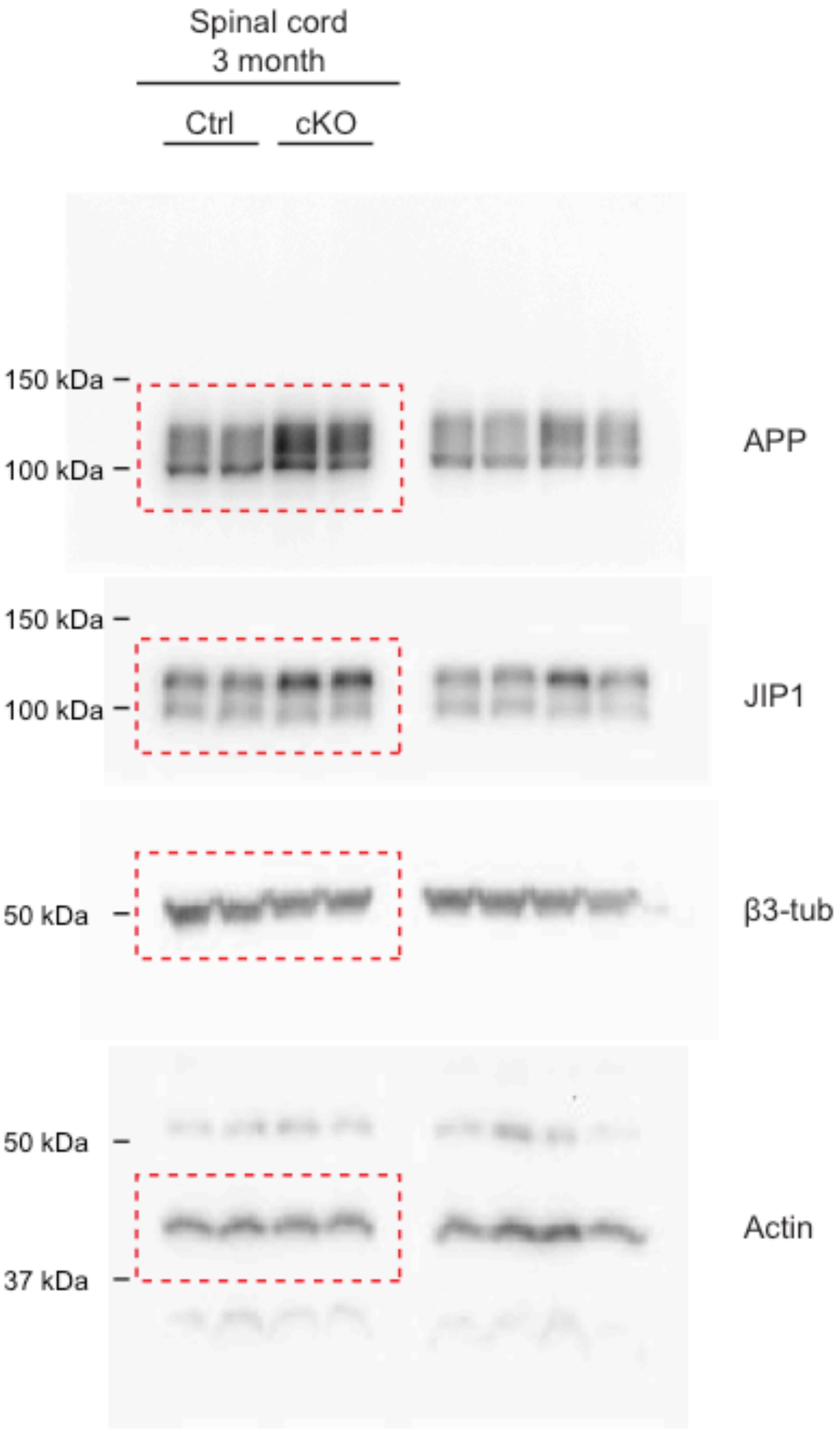

Supplementary Figure S11: Unprocessed scanned images of Figure 6b.

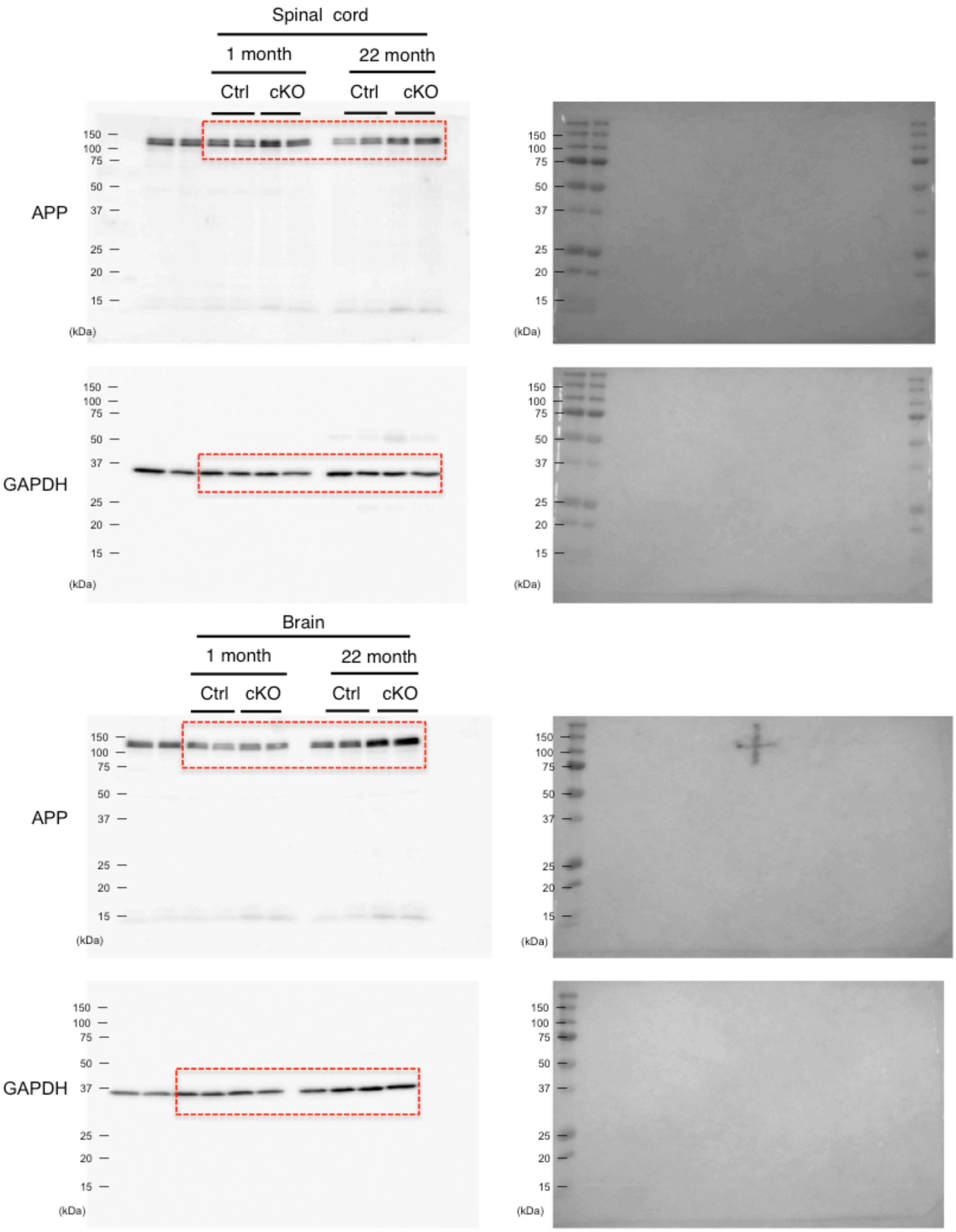

Supplementary Figure S12: Unprocessed scanned images of Figure 6c and 6d.

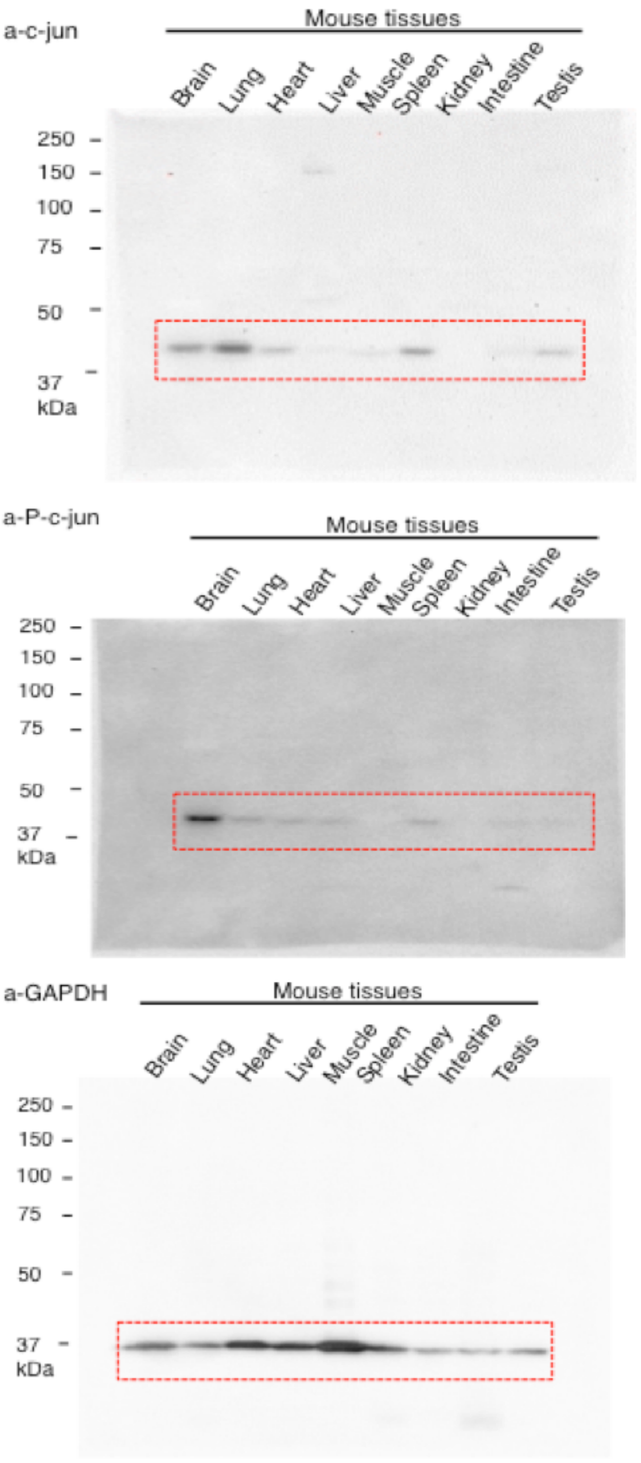

Supplementary Figure S13: Unprocessed scanned images of Figure S2.

Supplementary video 1

6 month-old control mouse.

Supplementary video. 2

6 month-old *Mkk7*$^{flox/flox}$ *Syn-Cre* mouse.

Supplementary video. 3

20 month-old control mouse.

Supplementary video. 4

20 month-old *Mkk7*$^{flox/flox}$ *Syn-Cre* mouse.

References

1. Yamasaki, T. *et al.* Stress-activated protein kinase MKK7 regulates axon elongation in the developing cerebral cortex. *J. Neurosci.* **31**, 16872–16883 (2011).
2. Okamoto-Uchida, Y. *et al.* The mevalonate pathway regulates primitive streak formation via protein farnesylation. *Sci. Rep.* **6**, 37697 (2016).
